# The efficacy of drug induced sleep endoscopy using multimodality monitoring system

**DOI:** 10.1371/journal.pone.0209775

**Published:** 2018-12-31

**Authors:** Hyung Chae Yang, Eun Kyung Jung, Sung Ho Yoon, Hyong-Ho Cho

**Affiliations:** Department of Otolaryngology-Head and Neck Surgery, Chonnam National University Medical School and Chonnam National University Hospital, Gwangju, South Korea; IRCCS Istituto Delle Scienze Neurologiche di Bologna, ITALY

## Abstract

**Introduction:**

To overcome the limited readability of the conventional drug induced sleep endoscopy (DISE) system which only records flexible endoscopy images, we devised the Multimodality DISE System (MDS). MDS encompasses the monitoring systems for oxygen saturation, electrocardiogram, blood pressure, snoring intensity, and patient’s position. It enabled to record comprehensive situation of patients who underwent DISE. In this study, we compared the efficacy of MDS with that of the conventional DISE system.

**Methods and materials:**

Ten patients underwent DISE at a tertiary hospital. DISE evaluated the airway of each patient in four positions; supine, supine with jaw thrust, right lateral decubitus, and left lateral decubitus. In addition, every examination was recorded by using both single monitoring system and MDS system. Five otolaryngologists interpreted the recorded examinations without knowledge of patient information (10 conventional DISE and 10 MDS). The visual analogue scale (VAS) scores for readability, reading times, ease of patient explanation and the ease of decision making were analyzed.

**Results:**

Mean VAS scores for readability of conventional DISE and MDS were 4.41+2.56 and 8.42+2.07 (p<0.001). Mean reading times for conventional DISE and MDS were 238.80+61.26 sec and 81.00+44.99 sec, respectively (p<0.001). MDS showed superiority in patient communication (p<0.001). MDS was helpful in decision making regarding patients with obstructive sleep apnea (p<0.001).

**Conclusions:**

MDS enhanced the readability of previously recorded DISE and enabled easier doctor-patient communication. In addition, MDS is more effective in decision making regarding patients with OSA. MDS has laid the groundwork for separating the DISE prescriber from the DISE performer.

## Introduction

Ever since drug-induced sedation endoscopy (DISE) was first introduced in 1991 [1], it has become one of the most widespread techniques to assess the airway and the anatomy of patients with snoring and obstructive sleep apnea (OSA) [2]. Before the advent of DISE, several methods such as Muller maneuver, lateral cephalometry, and computed tomography (CT) were used to evaluate the airway [1]. However, these methods could not evaluate the airway during sleep [1, 3]. The ability to visualize the site of obstruction during sleep is the strongest feature of DISE [1–4]. The validity and the reliability of DISE were ascertained in many studies, and recently, European position paper on DISE was published [2, 5].

During DISE examination, the physician not only analyses the airway in the supine sleep position. Various body positions and situations such as lateral sleep position, head turning, mandibular pull up, mouth opening, or continuous positive airway pressure (CPAP) titration can be applied [5–7]. These simulations provide crucial information for decision making [3, 8, 9]. However, the conventional single channel DISE system usually records flexible endoscopic images only. Based only on the recorded video clips with the conventional DISE system, it is difficult to become aware of the surrounding information [7]. Without surrounding information, position change of patients or applied maneuver cannot be discriminated, not to mention the vital signs of patients. As a result, the physician usually performs DISE for his patients by himself. It is not easy to separate the prescriber from performer [10, 11]. In addition, simultaneous monitoring and examination of patients is also not easy. These are the biggest limitations of DISE in practice.

To overcome the limitations of the conventional single modality DISE system, we devised the Multimodality DISE System (MDS). It encompasses the endoscopic monitoring, and display systems for oxygen saturation, electrocardiogram, blood pressure, snoring intensity, and patient’s position. We hypothesized that comprehensive monitoring and recording of patients and surrounding situation could enhance the efficacy and readability of DISE. Therefore, in this study, we compared efficacy of single modality DISE and MDS.

## Methods and materials

### Patients

This study was a single-institution, randomized cross-over trial. Ten patients were enrolled. They underwent DISE between July 2015 and February 2016 at a tertiary hospital for evaluation of snoring and sleep apnea. Eligibility criteria for study enrollment were adults, aged over 18 years with snoring or apnea hypopnea index (AHI) over 5. Patients with age below 18 years, side effects of sedative drugs, or who failed the examination for any reason were excluded. All DISE examinations were recorded by using MDS system. The Institutional Review Board of the Chonnam National University Hospital approved this study protocol (#CNUH-2017-156).

### DISE protocol

**Fig 1:**
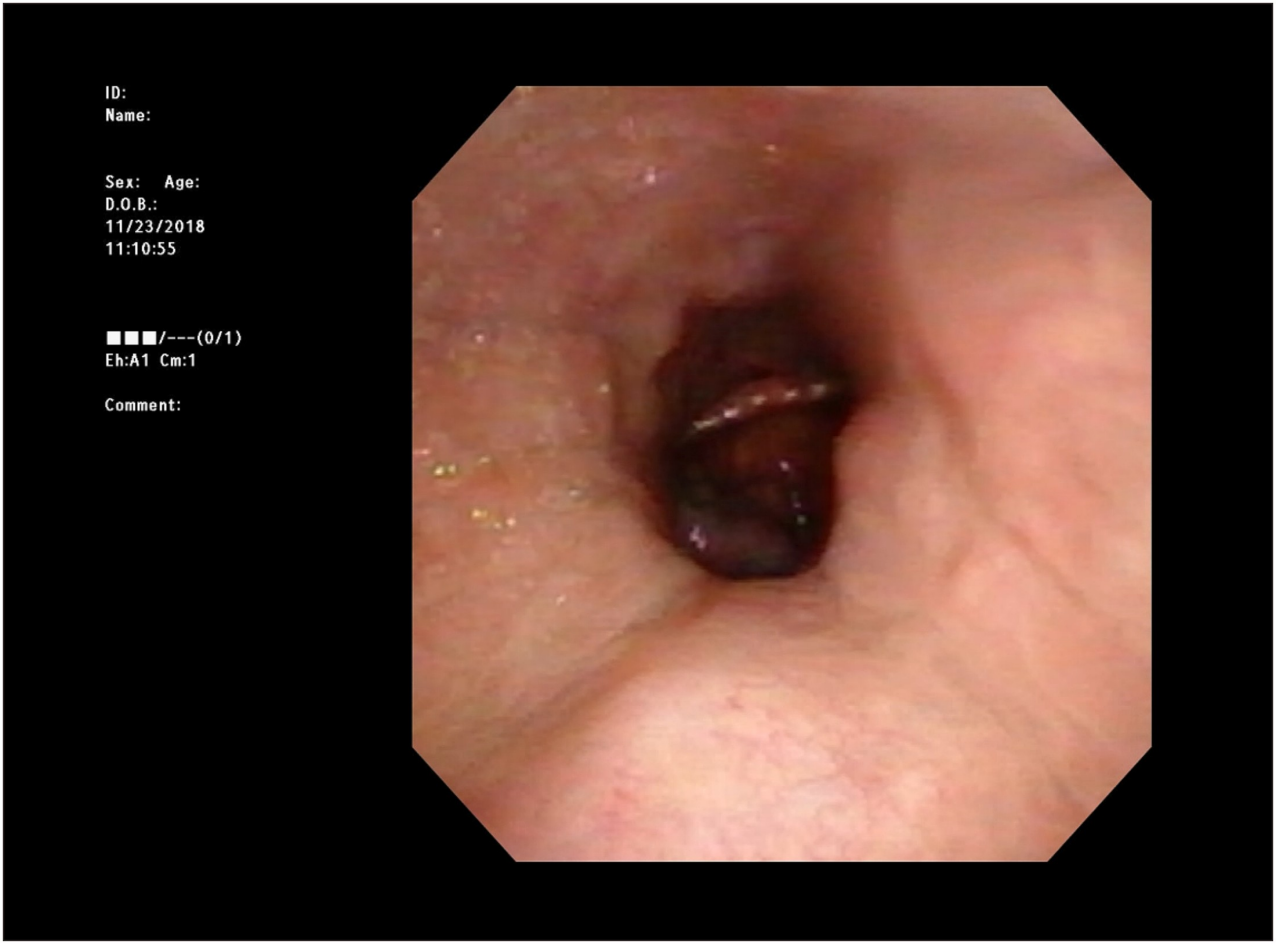
Captured image of conventional drug induced sleep endoscopy system (DISE). A conventional single channel DISE system records flexible endoscopic images only. It does not provide any surrounding information except endoscopic finding. However, surrounding information such as oxygen saturation, position of patients, appliance of continuous positive airway pressure (CPAP), or pressure of CPAP are crucial to understand the examination.

All DISE examinations were performed in the operation room using standard vital monitoring systems such as oxygen saturation, electrocardiogram, and blood pressure. The examination evaluated the pattern of airway obstruction in each patient in four positions; supine, supine with jaw thrust, right and left lateral decubitus, in sequential order. In addition, every examination was recorded by the MDS (Fig 1). IV dexmedetomidine and propofol were used as sedative agents, alone or in combination. Bispectral index (BIS) values were monitored whenever the system was available, maintaining the sedation level from 60 to 70.

### MDS system

**Fig 2:**
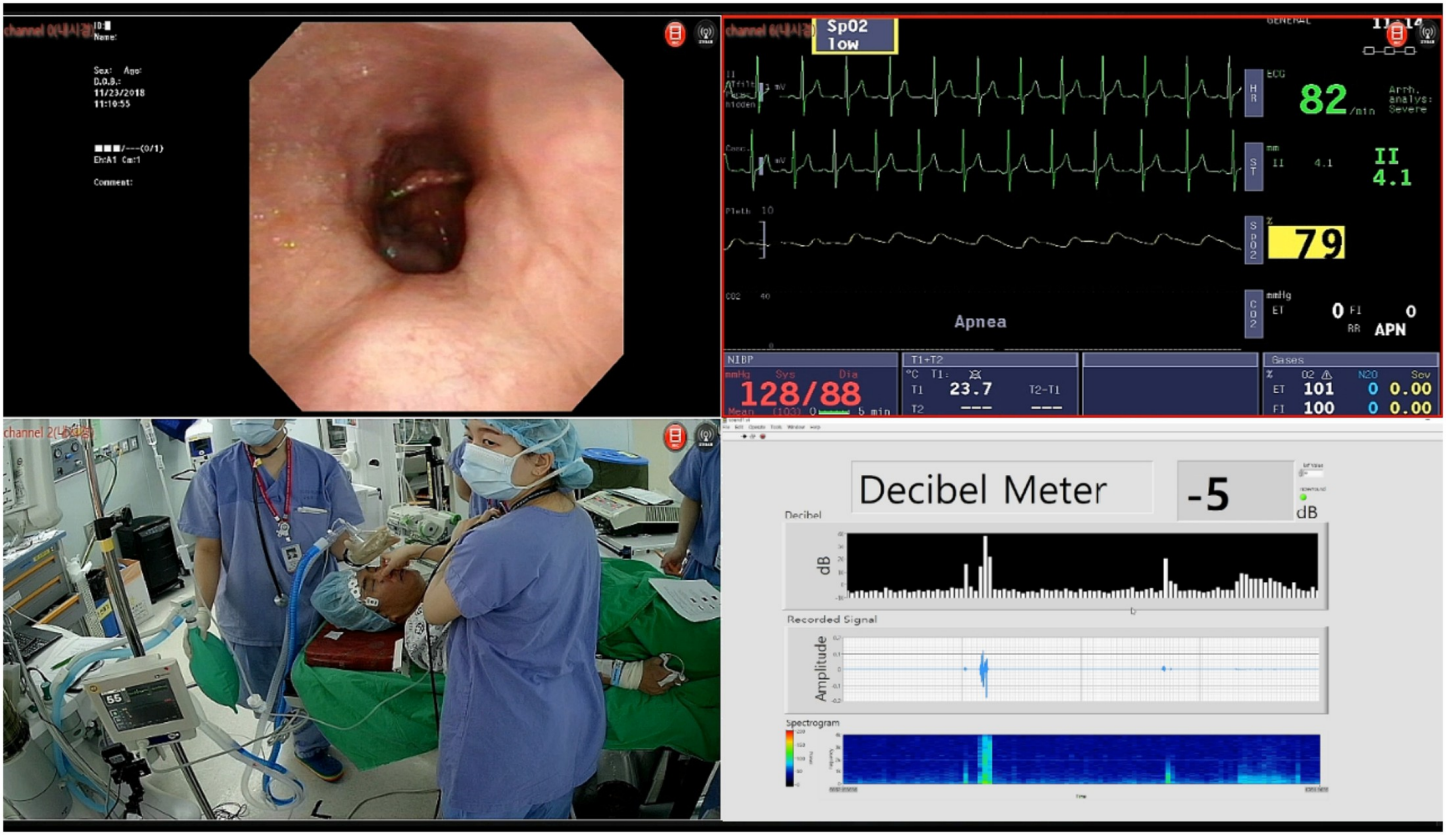
Captured image of Multimodality DISE System (MDS). Image features MDS recording during the examination. Upper left, recorded image of concentric collapse at soft palate. In counter clockwise direction, recorded images of external camera, sound analyzer, and vital monitor. Patient have concentric soft palate collapse (upper left) in supine position without mandibular pull up maneuver, at bispectral index level 62 (lower left). The level of pulse oximetry was decreased to 74% (upper right).

The MDS system integrates 4 or more independent systems; the flexible fiber endoscopy system, the vital monitoring system, the external monitoring camera system, and sound analyzer (Fig 2, Korean patent # 10–1716405). The video capture system of MDS is able to record 4 channels separately. After recording the examination, MDS can provide the recorded examination in a conventional single channel form (Fig 1 and S1 Video) or a MDS form (Fig 2 and S2 Video) as needed. Thus, SCD and MDS images can be acquired for the same examination.

### Interpretation

Five otolaryngologists who had experience of more than one hundred DISE examinations participated in this study. Each otolaryngologist interpreted 20 examinations of 10 patients; 10 SCD and 10 MDS. Twenty videos containing 10 single monitored DISE and 10 MDS examinations were given to 5 interpreters in a random order without knowledge of patient information. A questionnaire containing 7 questions about the ease in the aspects of readability, patient explanation, and decision making was administered to the panel members (S1 Table). Answers were checked using the visual analogue scale (VAS) score. In addition, the time required for reading each examination were recorded.

### Statistical analysis

The paired t-test was used to compare continuous variables answered in the questionnaire, which was about the efficacy of DISE in deciding the treatment plan and the readability of single modality DISE and MDS. All statistical analyses were performed using IBM SPSS Statistics for Windows ver 20.0 (IBM Corp, Armonk, NY). Two-sided p values <0.05 were considered statistically significant.

## Results

A total of 10 patients were enrolled in this study. Median age was 51.8 years, ranging from 33 to 67 years, and mean AHI was 19.31 events/hour. Demographics and baseline characteristics of patients are described in Table 1. Six patients underwent DISE with use of dexmedetomidine for sedation, while the other 4 patients were administered propofol.

**Table 1:** Demographics of the study population*.

Total number of patients included in study		10
Gender	Male	9
	Female	1
Median Age	years	51.8 (33–67)
Median BMI	kg/m^2^	26. 27 (21.12–30.43)
Median AHI	events/hour	19.31 (2.4–40)
Median Minimal SaO2	%	85.2 (77–94)
Administered Drug	Dexmedetomidine	6
	Propofol only	4

* BMI denotes body mass index; AHI, Apnea hypopnea index: SaO2, oxygen saturation

Mean VAS scores for readability and ease of patient explanation were significantly increased (p<0.001). The time required for reading the DISE examinations was significantly reduced with use of the MDS system (p<0.001, Table 2). There was no significant difference in the likelihood of oral appliance recommendation according to the type of DISE (p = 0.140). However, MDS was helpful in decision making regarding oral appliance prescription, lateral sleep recommendation (p<0.001), and overall treatment decision making (p<0.001). In addition, lateral sleep position was more frequently recommended in MDS group (p = 0.048, Table 3).

**Table 2:** Readability of drug induced sleep endoscopy according to the monitoring system *.

	Conventional DISE system (n = 10)	MDS (n = 10)	*p*-value^†^
Patient explanation	5.76 ±2.62	8.27 ± 1.97	<0.001
Readability	4.41 ± 2.56	8.42 ± 2.07	<0.001
Reading time (min)	238.80 ± 61.26	81.00 ± 44.99	<0.001

* Values were scored using 10 cm visual analogue scale (VAS).

Plus-minus values are means ± standard deviation.

MDS denotes multimodality drug induced sleep endoscopy system; MAD, mandibular advancement device.

^†^ The paired t-test was used to compare VAS scores between conventional DISE and MDS.

**Table 3:** The effect of DISE on decision making according to the monitoring system *.

	Conventional DISE system (n = 10)	MDS (n = 10)	*p*-value^†^
How strongly recommend MAD?	4.56 ± 3.53	5.40 ±3.83	0.140
Self confidence in decision making regarding MAD	6.11 ± 2.79	7.93 ±2.09	0.000
How strongly recommend positional sleep	2.69 ± 3.05	3.71 ±3.45	0.048
Self confidence in decision making regarding positional therapy	5.24 ± 2.84	7.31 ±2.58	0.000
Helpfulness of DISE for overall decision making	5.86 ± 2.69	8.04± 2.06	0.000

* Values were scored using 10 cm visual analogue scales (VAS). Plus-minus values are means ± standard deviation. MDS denotes multimodality drug induced sleep endoscopy system; MAD, mandibular advancement device

^†^ The paired t-test was used to compare VAS scores between conventional DISE and MDS.

## Discussion

To overcome the limitations of the conventional DISE system with single modality monitoring, we developed the multimodality monitoring DISE system. In order to increase the readability, we equipped the multi-channeled system and split one large display into multiple displays. As shown in Fig 1, conventional DISE displayed only the endoscopic image of the patient’s airway. However, MDS could provide more information such as oxygen saturation, heart rate, ECG, Blood pressure, patient’s position, physician’s maneuver, and/ or snoring intensity simultaneously (Fig 2). The types of display can be changed according to the demand of each physician.

External camera placed on the display monitor can provide information regarding the patient’s position such as supine, lateral head turning, and jaw thrust maneuver during DISE. Based on this result, MDS may achieved significantly higher efficacy in decision making regarding prescription of oral appliance or recommendation of lateral sleep position. In addition, with use of MDS, the physician could detect an unintentionally missed procedural sequence. Interestingly, during the study, we unintentionally omitted the jaw thrust maneuver in one patient. With use of SCD, 4 out of the 5 physicians could not detect the missed sequence. On the contrary, 4 out of the 5 physicians could detect the missed sequence with use of MDS.

Communication with patients can be improved with use of the MDS system. It is important in practice. Drug used for DISE, such as propofol or midazolam, has an anterograde amnesic effect [5, 12, 13]. The patients tended to forget the conversation even after they were awakened. As a result, physicians frequently requested for an explanation on the result of DISE examination during their next visit. In this situation, MDS was useful for both the doctor and the patient. MDS enabled us to obtain the endoscopic findings with surrounding information. The author was able to genuinely experience high satisfaction among the patients due to visual supplements, resulting in better understanding in lesser amount of time in the outpatient department. This fact might have influenced the high VAS score for doctor-patient communication.

MDS may affect the pattern of DISE practice. First, DISE can be ordered like other radiologic examination such as computed tomography or magnetic resonance images. MDS provides sufficient information for understanding the surrounding situation during DISE only with recorded video clips. A clinician does not need to perform DISE by himself in the operation theater or an equipped room. Second, if the clinician can order DISE for a patient, it will be performed more widely. One can eliminate many kind of risk may be caused by sedatives endoscopy. In addition, the clinician can save time and space required for DISE examinations.

To overcome the limitations of the conventional DISE system and to maximize the potential of DISE, we devised the MDS. By recording much more essential information at the same time, MDS can achieve better readability, lesser reading time, and improvement of decision making. In addition, MDS can play a key role in a widespread usage of DISE since it can provide the prescriber with all pivotal information about the performer experience.

Although we draw statistically significant conclusions, the limited number of patients is a limitation of this study. Future studies with a large number of subjects are needed to confirm the results.

## Supporting information

S1 Table Questionnaire for evaluating the efficacy of drug induced sleep endoscopy systems. (DOCX)

S1 Video A video recording of the conventional drug induced sleep endoscopy system. (ZIP)

S2 Video A video recording of the conventional drug induced sleep endoscopy system. (ZIP)

S1 File Dataset of the study. (XLSX)
