# Supplementary material for: The efficacy of drug induced sleep endoscopy using multimodality monitoring system
Source: PLoS One. 2018 Dec 31;13(12):e0209775. doi: 10.1371/journal.pone.0209775 (PMC6312210; doi:10.1371/journal.pone.0209775)
Supplement: S1 Table — (DOCX) [file pone.0209775.s001.docx]

# Supporting information

S1 Table. Questionnaire for evaluating the efficacy of drug induced sleep endoscopy systems. *

| Questionnaire (for question 4 and 6, irrelevant for recommending or nonrecommending, if DISE helps in determining the treatment method, the score is close to 10. Otherwise, the score is close to 0). |
| --- |
| 1. Is DISE recording readable? 2. Is DISE recording useful for explaining to the patient? 3. How strongly will you recommend MAD (oral appliance) to the patient? |
| 1. Are you certain about recommending MAD? |
| 1. How strongly will you recommend positional treatment to the patient? |
| 1. Are you certain about recommending positional therapy? |
| 1. Does the DISE result help in making the general treatment plan? |

* DISE denotes drug induced sleep endoscopy; MAD mandibular advancement device.
